# A rare case report of adenoid cystic carcinoma of parotid gland with perivascular and perineural spread

**DOI:** 10.1259/bjrcr.20210249

**Published:** 2022-02-24

**Authors:** Divya Pabbisetti, Bala Subrahmanyam Praharaju, Jyothik Varun Inampudi, Prudviram Jakkireddy, Vamsi Machineni, Anantaram Gudipati, Amber Papalkar, Chaithanya Isamalla

**Affiliations:** 1 Department of Radiology and Imageology, Krishna institute of medical sciences, Minister Road, Begumpet, Secunderabad, Telangana, India; 2 Siddhartha Medical College, Dr.NTR University of Health Sciences, Vijayawada, Andhra Pradesh, India

## Abstract

Adenoid cystic carcinoma is a slow growing malignant neoplasm of the salivary glands, mainly occurring in minor salivary glands and relatively rare in parotid glands. It has got a remarkable capacity for extensive subclinical spread to the adjacent structures. Here, we present a case report of adenoid cystic carcinoma of parotid gland with perineural and perivascular spread. To our knowledge, this is the first reported case of adenoid cystic carcinoma of parotid gland with perivascular spread along a major artery (ECA) and perineural spread along two different nerves (mandibular nerve and facial nerve)

## Description

A 56-year-old male patient, with history of lower motor neuron type of facial palsy and swelling over the right angle of mandible from 8 months now presented with chief complaints of pain and increase in the size of swelling since 2 months.

**Figure 1.**
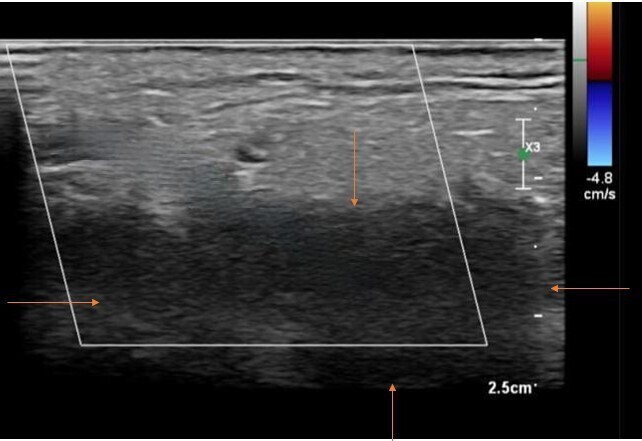
USG of right parotid gland showed a well defined irregular hypoechoic lesion in superficial and deep lobes (indicated by orange arrows) No significant vascularity is noted within the lesion in doppler study

**Figure 2.**
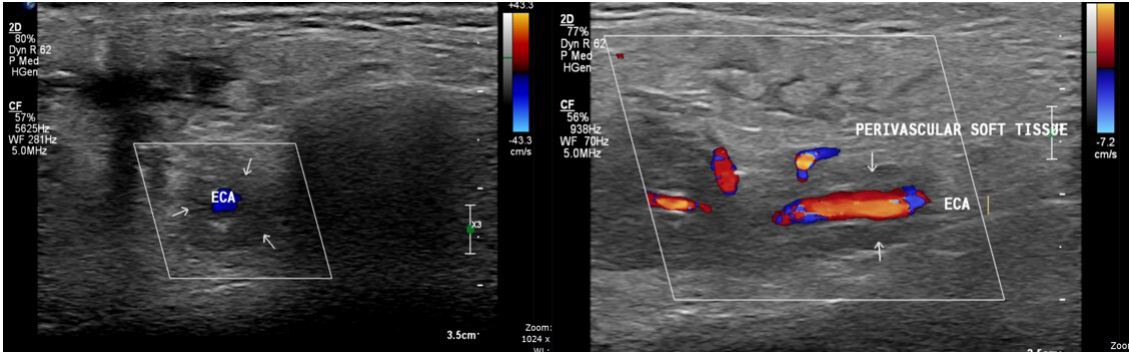
The tumour extends along the external carotid artery forming a soft tissue cuff around it beyond the confines of the parotid gland

On examination, facial asymmetry and swelling over the right parotid region were present. There was no deviation of tongue. Vision was normal. The patient was referred for ultrasonography of the right parotid region which showed a well-defined irregular hypoechoic lesion in superficial and deep lobes of right parotid gland.Figure 1 The lesion extends along the external carotid artery forming a soft tissue cuff around it beyond the confines of the parotid gland.Figure 2

**Figure 3.**
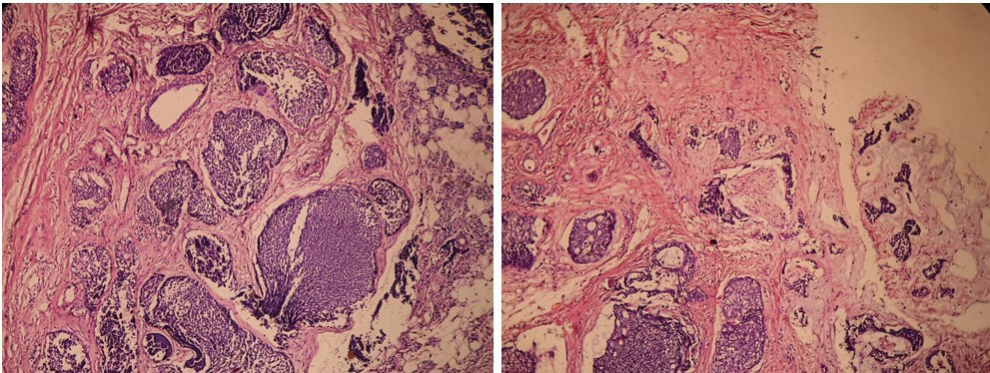
Photomicrographs showing features of adenoid cystic carcinoma with predominant solid pattern and perineural spread

Excision biopsy of the intraparotid portion of the tumor was done and the specimen was sent for histopathological examination which showed features of adenoid cystic carcinoma with predominant solid pattern with focal cribriform pattern and perineural spread.Figure 3

**Figure 4.**
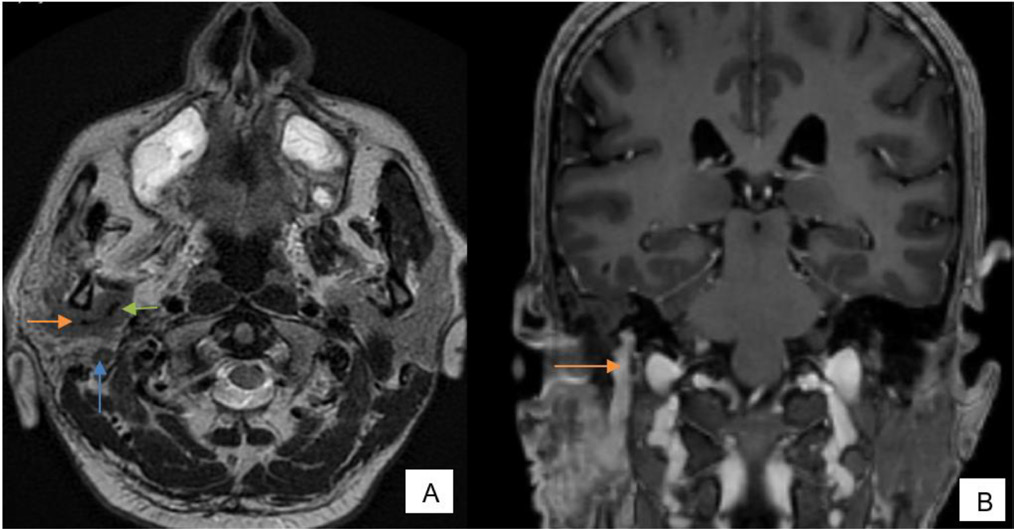
(**A**) FSE T2 axial—well-defined mass lesion in superficial and deep lobes of right parotid gland encasing terminal branches of ECA, *i.e.* superficial temporal artery (orange arrow) and maxillary artery (green arrow). Tumor extending along intraparotid portion of facial nerve (blue arrow). Fatty atrophy of right sided muscles of mastication (**B**) Post-contrast SPGR coronal—intensely enhancing tumor extending along mastoid segment of right facial nerve (orange arrow). FSE, fast spin echo.

**Figure 5.**
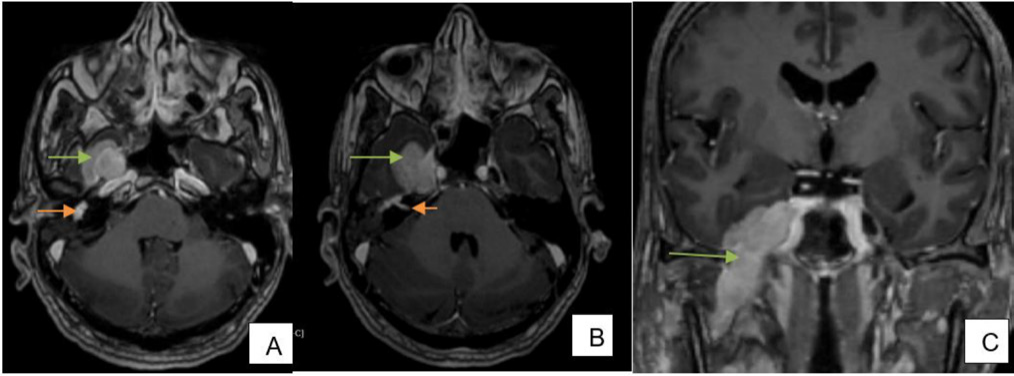
(**A**) Post-contrast SPGR axial—intensely enhancing tumour extending along the tympanic segment of right facial nerve (orange arrow)—perineural spread along facial nerve. Intensely enhancing tumour at enlarged right foramen ovale (green arrow)—perineural spread along mandibular nerve. (**B**) Post-contrast SPGR axial- intensely enhancing tumour extending along the canalicular segment and anterior genu of right facial nerve (orange arrow)—perineural spread along facial nerve. Intensely enhancing tumor involving right Meckels cave with extra-axial component indenting right medial temporal lobe (green arrow)—perineural spread along mandibular nerve. (**C**) Post-contrast SPGR coronal—intensely enhancing tumor along the mandibular nerve in masticator space through grossly widened foramen ovale into Meckels cave indenting the adjacent right medial temporal lobe (green arrow)—perineural spread along mandibular nerve

**Figure 6.**
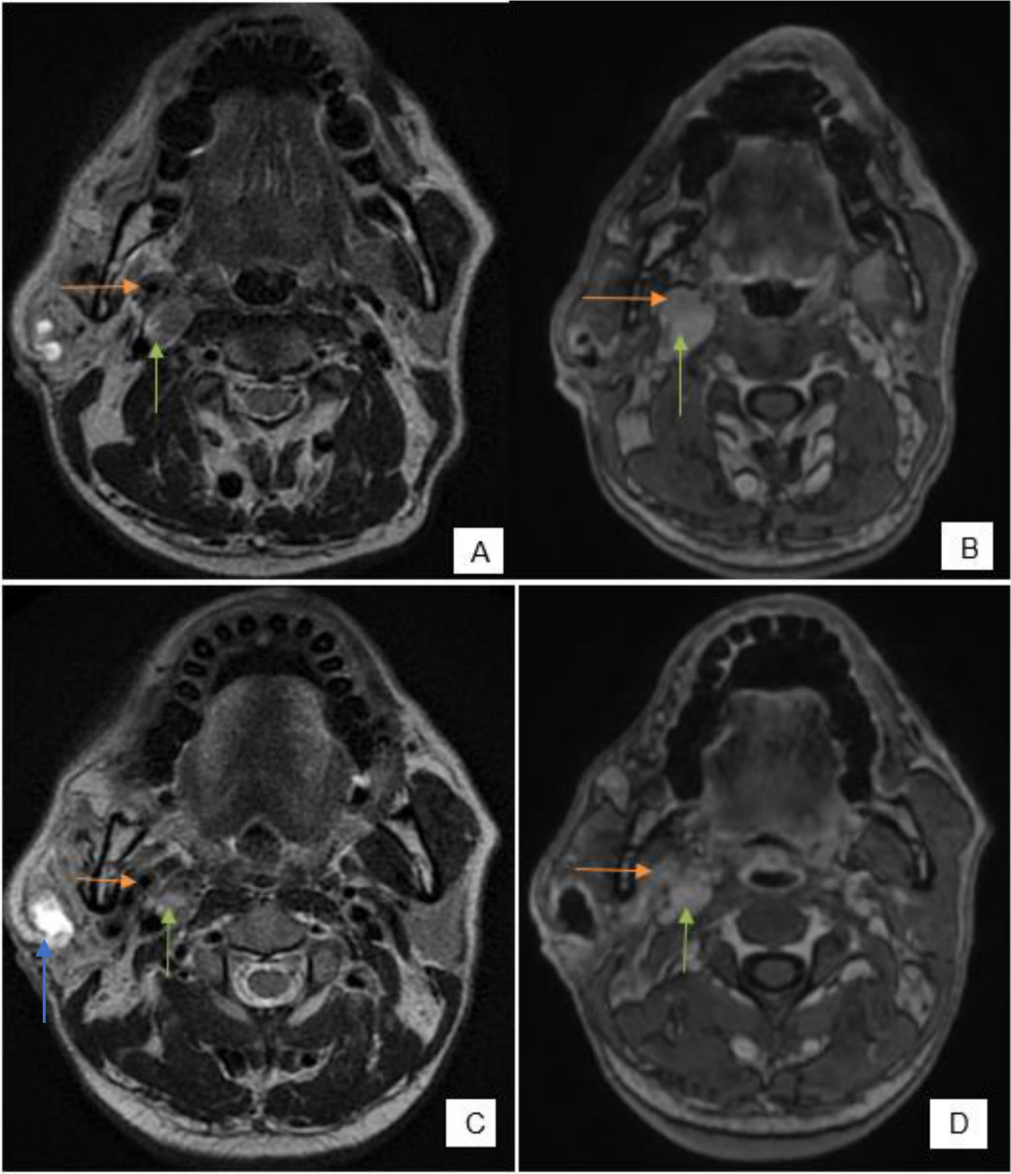
(**A, C**) FSE T2 axial, (**B, D**) Post-contrast SPGR axial, C and D are few sections superior to A and B—intensely enhancing soft tissue cuff along the right ECA (orange arrow). Intensely enhancing discrete lesion in right carotid space—lymph nodal deposit (green arrow). Blue arrow in C indicates the post biopsy cavity in right parotid gland. FSE, fast spin echo.

**Figure 7.**
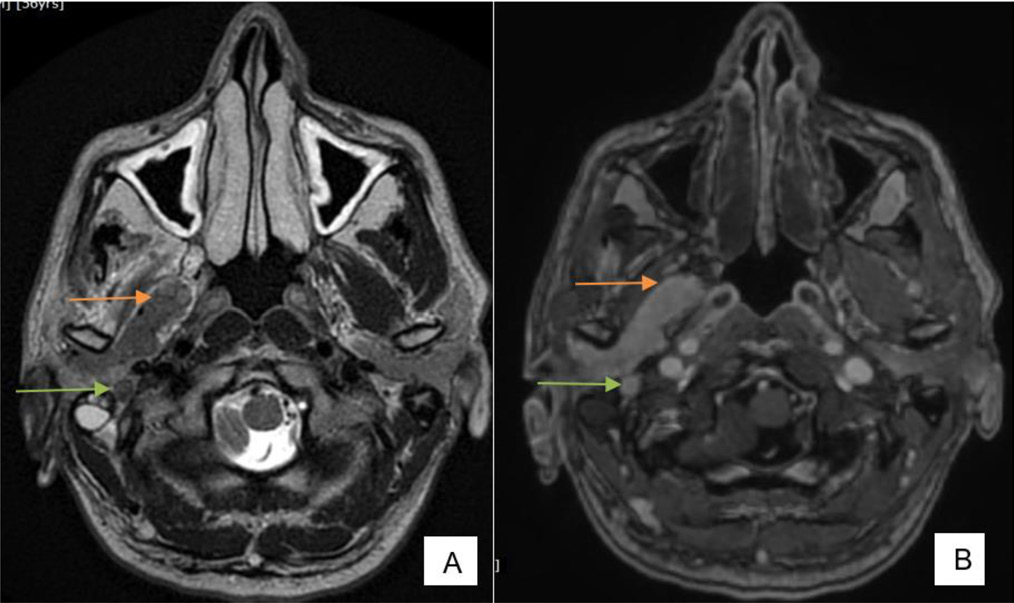
(**A**) FSE T2 axial, (**B**) Post-contrast SPGR axial—nodular extension of the tumor into right masticator space (orange arrow)—perineural spread along the branches of mandibular nerve, tumor extending along mastoid segment of facial nerve (green arrow). FSE, fast spin echo.

**Figure 8.**
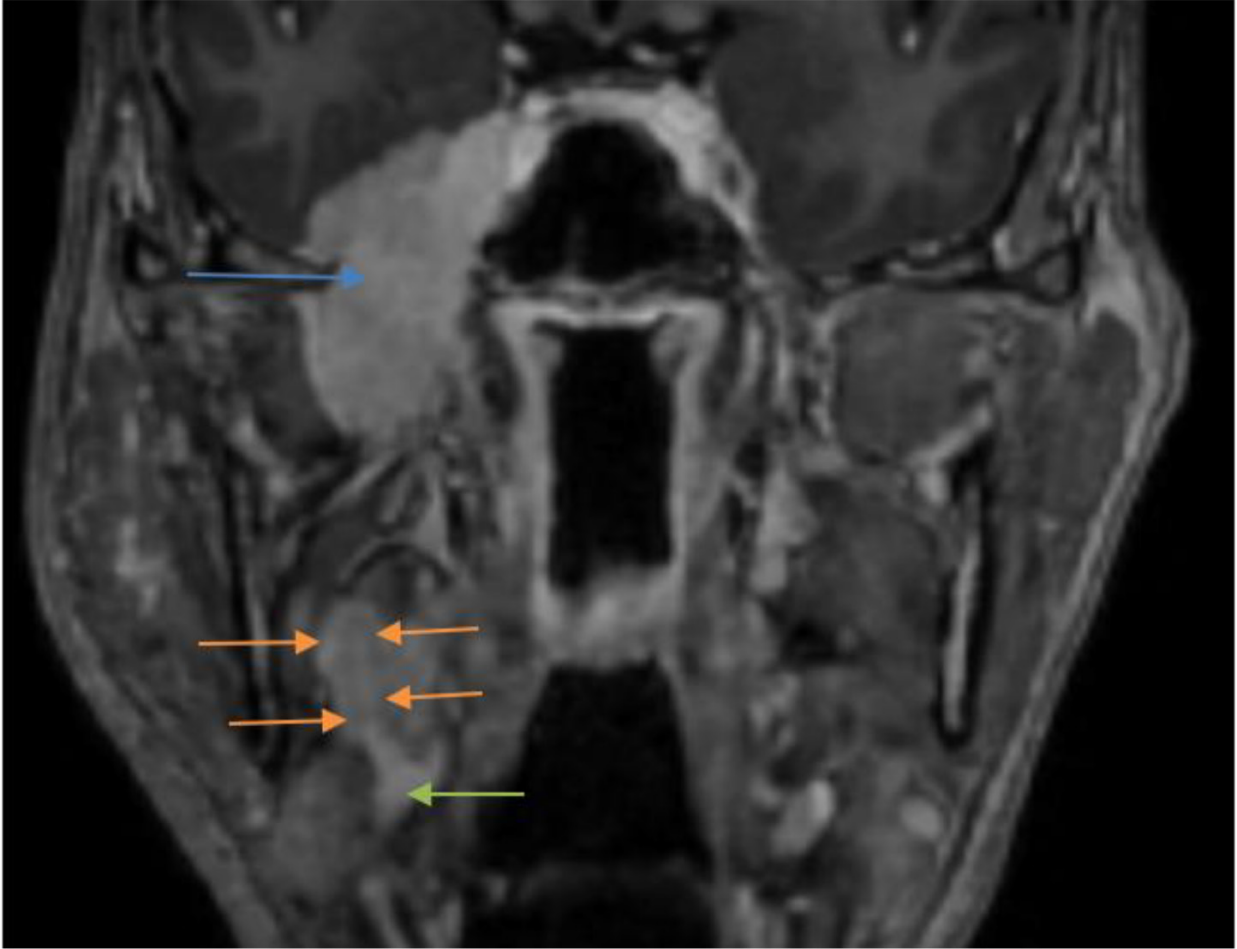
Post-contrast SPGR coronal—intensely enhancing tumor along the right ECA (orange arrows)—perivascular spread. Right CCA bifurcation is labeled with green arrow. Perineural tumor component along right mandibular nerve through widened foramen ovale is labelled with blue arrow.

Further imaging evaluation with MRI neck was done which showed a well-defined T1/T2 hypointense lesion involving superficial and deep lobes of right parotid gland. The lesion is completely encasing the intraparotid portion of the right external carotid artery and its terminal branches, *i.e.* maxillary artery and superficial temporal artery. A cuff of tumor is also extending along the right ECA beyond the confines of the parotid gland- suggestive of perivascular spread. Nodular extension of the lesion into the masticator space is noted, extending along the mandibular (V3) division of right trigeminal nerve through the grossly widened right foramen ovale with right temporal extraaxial component causing indentation on right medial temporal lobe. Posteriorly, the lesion is extending into the Meckel’s cave and cavernous sinus—suggestive of perineural spread along mandibular (V3) division of trigeminal nerve. Fatty atrophy of right-sided muscles of mastication and muscles of floor of mouth. The lesion is also extending along parotid, mastoid, tympanic, canalicular segments of the right facial nerve and greater superficial petrosal nerve—suggestive of perineural spread along facial nerve. The lesion, its perivascular and perineural extensions show intense enhancement on post-contrast study. Intensely enhancing nodular lesion is noted in right carotid space discrete from the above-mentioned lesion splaying right ECA and ICA which is suggestive of a lymph nodal deposit.Figures 4–8

The patient underwent positron emission tomography CT which did not reveal any distant metastasis

### Final diagnosis

– Adenoid cystic carcinoma of right parotid gland with perivascular spread along external carotid artery and perineural spread along mandibular (V3) division of trigeminal nerve and along facial nerve

Due to the inoperable stage of the tumor, radiotherapy was considered.

## Discussion

Adenoid cystic carcinoma (ACC) is a salivary gland neoplasm with aggressive nature and slow growth. They constitute about 21.9 % of all salivary gland malignancies including major and minor salivary glands. It has got a remarkable capacity for recurrence.^1,2^ It can arise in any salivary gland, but mainly develops within the minor salivary glands and submandibular gland. In the parotid gland, ACC is relatively rare, accounting to about 2–3% of all tumors^3^ . In head and neck region, some of the sites of origin are the tongue, larynx, paranasal sinuses, nasopharynx, lacrimal glands and external auditory canal. They occur mainly among females, between fifth and sixth decades of life.

As compared to its aggressive nature, the clinical presentation is rather indolent which belies its extensive subclinical spread to the adjacent structures. It presents as a slow growing mass with pain, facial nerve paralysis when involving the parotid gland and local invasion. They are locally aggressive with perineural and perivascular spread with late distant metastases.^4^ Perivascular invasion has been reported in some cases of ACC and was found to be associated with a higher rate of metastasis. To our knowledge, this is the first reported case adenoid cystic carcinoma of parotid gland with perivascular spread along a major artery (ECA) and perineural spread along two different nerves (mandibular nerve and facial nerve).

Adenoid cystic carcinoma is histologically characterized by three patterns—cribriform, tubular and solid.^5^ Most of the tumors show a mix of these patterns. Mitotic figures are more commonly visualized in solid patterns which have the worst prognosis.

ACCs are categorised into three grades. There are no solid areas in Grade I which is composed of cribriform and tubular patterns. Solid areas are seen in Grade II (less than 30 ) and Grade III (more than 30 percent). Atypical cells and mitoses are seen in Grade III^6^ . Our case showed a predominantly solid pattern (more than 30%), thereby fitting into Grade III.

Metastasis to lungs is most common followed by liver. Unlike most malignancies, lymph nodal metastasis is rare.

Various theories for the underlying mechanism of perineural spread of the tumor have been hypothesized like propagation of tumor along lymphatics, theory of least resistance pathway provided by nervesand role of neural cell adhesion molecules^7^ .

Vascular endothelial growth factor (VEGF) overexpression has been seen in some tumors showing perivascular spread.^8^ One possible explanation is that the VEGF secreted by the tumor cells gets attached to VEGF receptors in the blood vessel wall leading to perivascular tumor growth.

The mainstay of treatment is surgical, while radiotherapy can be considered for advanced stages and as an adjuvant to total surgical resection. As lymph nodal metastasis to regional lymph nodes is uncommon, neck dissection is usually not indicated.^3^ Advanced and non-resectable tumors may be treated only with radiotherapy.

## Conclusion

Adenoid cystic carcinoma is a slow growing secretory gland neoplasm with a remarkable capacity for extensive local spread and recurrence. It has got a propensity for perivascular and perineural spread. Definitive treatment is surgical and radiotherapy can be considered for advanced cases or as an adjuvant.

## Learning points

- Adenoid cystic carcinoma of the salivary glands is an aggressive slow growing tumor which mainly occurs in minor salivary glands and rarely in parotid gland.
- In every case of parotid tumor, we must consider the possibility of adenoid cystic carcinoma and actively look for perineural and perivascular spread as they have management and prognostic implications
- Treatment should be aimed at local disease control, improving function and preventing distant metastasis for better prognosis and improving the quality of life.
